# Supplementary material for: Association between oral microbiome and seven types of cancers in East Asian population: a two-sample Mendelian randomization analysis
Source: Front Mol Biosci. 2023 Nov 21;10:1327893. doi: 10.3389/fmolb.2023.1327893 (PMC10702768; doi:10.3389/fmolb.2023.1327893)
Supplement: Supplementary file 1 [file DataSheet1.ZIP › Supplementary Materials/MR plots of saliva/saliva═╝/Lung Cancer/pheno.2346_to_lung cancer_leave_one_out.pdf]

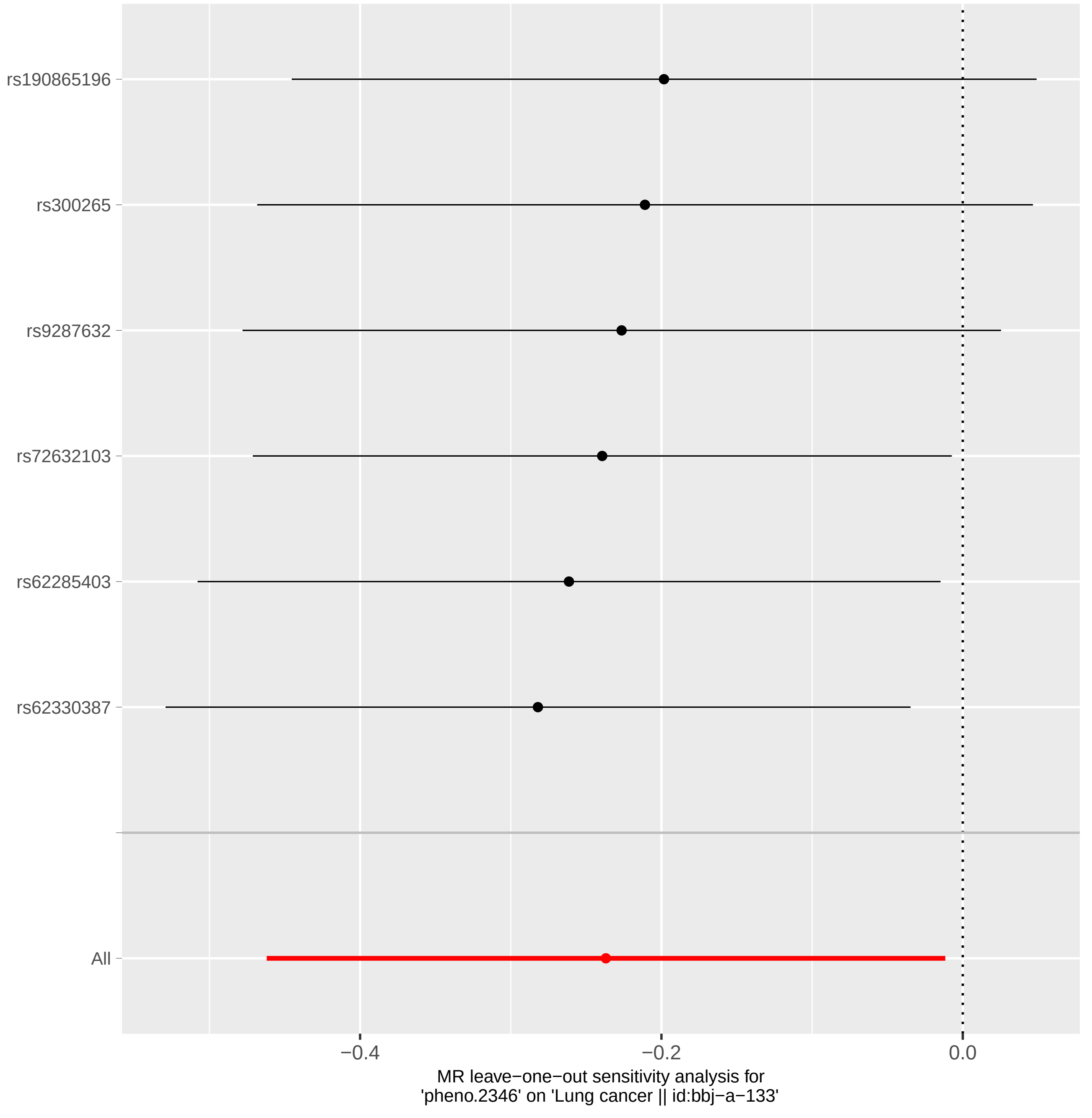
rs190865196
rs300265
rs9287632
rs72632103
rs62285403
rs62330387
All
−0.4
−0.2
0.0
MR leave−one−out sensitivity analysis for
'pheno.2346' on 'Lung cancer || id:bbj−a−133'
